# Stand Out in Class: restructuring the classroom environment to reduce sedentary behaviour in 9–10-year-olds — study protocol for a pilot cluster randomised controlled trial

**DOI:** 10.1186/s40814-018-0295-3

**Published:** 2018-05-24

**Authors:** Stacy A. Clemes, Daniel D. Bingham, Natalie Pearson, Yu-Ling Chen, Charlotte Edwardson, Rosemary McEachan, Keith Tolfrey, Lorraine Cale, Gerry Richardson, Mike Fray, Stephan Bandelow, Nishal Bhupendra Jaicim, Jo Salmon, David Dunstan, Sally E. Barber

**Affiliations:** 1 National Centre for Sport and Exercise Medicine, School of Sport, Exercise and Health Sciences, Loughborough University, Loughborough, UK; 2 NIHR Leicester Biomedical Research Centre, Leicester, UK; 3 Bradford Institute for Health Research, Bradford Teaching Hospitals Foundation Trust, Bradford, UK; 4 Diabetes Research Centre, University of Leicester, Leicester, UK; 5 Centre for Health Economics, University of York, Heslington, York, UK; 6 Loughborough Design School, Loughborough University, Loughborough, UK; 7 Leicester Clinical Trials Unit, University of Leicester, Leicester, UK; 8 Institute for Physical Activity and Nutrition, School of Exercise and Nutrition Sciences, Deakin University, Melbourne, Australia; 9 Baker IDI Heart and Diabetes Institute, Melbourne, Australia; 10 Mary MacKillop Institute for Health Research, Australian Catholic University, Melbourne, Australia

**Keywords:** Sitting, Standing, Children, Sit-to-stand desks, Schools, Children’s health, Education

## Abstract

**Background:**

Sedentary behaviour (sitting) is a highly prevalent negative health behaviour, with individuals of all ages exposed to environments that promote prolonged sitting. Excessive sedentary behaviour adversely affects health in children and adults. As sedentary behaviour tracks from childhood into adulthood, the reduction of sedentary time in young people is key for the prevention of chronic diseases that result from excessive sitting in later life. The sedentary school classroom represents an ideal setting for environmental change, through the provision of sit-stand desks. Whilst the use of sit-stand desks in classrooms demonstrates positive effects in some key outcomes, evidence is currently limited by small samples and/or short intervention durations, with few studies adopting randomised controlled trial (RCT) designs. This paper describes the protocol of a pilot cluster RCT of a sit-stand desk intervention in primary school classrooms.

**Methods/Design:**

A two-arm pilot cluster RCT will be conducted in eight primary schools (four intervention, four control) with at least 120 year 5 children (aged 9–10 years). Sit-stand desks will replace six standard desks in the intervention classrooms. Teachers will be encouraged to ensure all pupils are exposed to the sit-stand desks for at least 1 h/day on average using a rotation system. Schools assigned to the control arm will continue with their usual practice, no environmental changes will be made to their classrooms. Measurements will be taken at baseline, before randomisation, and at the end of the schools’ academic year. In this study, the primary outcomes of interest will be school and participant recruitment and attrition, acceptability of the intervention, and acceptability and compliance to the proposed outcome measures (including activPAL-measured school-time and school-day sitting, accelerometer-measured physical activity, adiposity, blood pressure, cognitive function, academic progress, engagement, and behaviour) for inclusion in a definitive trial. A full process evaluation and an exploratory economic evaluation will also be conducted to further inform a definitive trial.

**Discussion:**

The primary output of this study will be acceptability data to inform the development of a definitive cluster RCT designed to examine the efficacy of this intervention on health- and education-related outcomes in UK primary school children.

**Trial registration:**

ISRCTN12915848 (retrospectively registered, date registered 9 November 2016).

**Electronic supplementary material:**

The online version of this article (10.1186/s40814-018-0295-3) contains supplementary material, which is available to authorized users.

## Background

Technological advances and changes to our environment and lifestyle have led to increased time being spent in sedentary behaviours [1]. Sedentary behaviour (sitting, lying, and reclining and expending < 1.5 metabolic equivalents [METs] [2]) is ubiquitous in modern society, with individuals of all ages exposed to environments that promote prolonged periods of sitting. Whilst it is acknowledged that physical activity is beneficial to health, sedentary behaviour has also been shown to adversely affect health. For example, high levels of sedentary behaviour have been independently associated with type 2 diabetes, cardiovascular disease, cancer and premature mortality in adults [3, 4].

Sitting is the most prevalent behaviour exhibited during waking hours in children from the UK, accounting for over 65% of their waking time [5], with some children reportedly sitting for over 10 h/day (> 70% of waking hours) [6]. Adverse associations between sedentary behaviour and cardio-metabolic health risk markers (obesity, blood pressure, cholesterol, insulin) have been reported in children [7, 8]. Given this and as sedentary behaviours track throughout childhood into adolescence and adulthood [9], the reduction of sitting time in young people is pertinent for the primary and secondary prevention of chronic diseases that result from excessive sitting in adulthood [3, 4]. Furthermore, with the emergence of an increased cardio-metabolic health risk profile being observed in the first decade of life in some ethnic groups in the UK (for example, higher levels of fasting insulin and triglycerides have been observed in 9–10-years-old British South Asian children in comparison to white British children) [10], may suggest that these individuals could be more vulnerable to the adverse health effects of excessive sitting time. The early reduction of sedentary behaviour in children in such higher-risk groups could be an important strategy for reducing ethnicity-related health inequalities later in life.

Children are increasingly exposed to environments and social norms that encourage prolonged sitting; for example, because the school classroom is equipped with standard desks and chairs, children are expected to sit throughout most lessons. To counter the detrimental effects of prolonged sitting on children’s health [7], strategies are needed to reverse the trend of increasing levels of sedentary behaviour. A meta-analysis of interventions targeting reductions in children’s sedentary behaviour reported an overall decrease of 18 min/day of sitting and reductions in body mass index (BMI) [11]. Whilst most studies were school-based, sedentary behaviour was targeted via behaviour change strategies within non-school settings (i.e. screen time at home). No included studies employed changes to the classroom environment. Classrooms are conducive to high volumes of sitting, with children typically sitting for longer during school hours than non-school hours [12]. Environmental change within the classroom is therefore an obvious means to target children’s sitting time and may address health inequalities in that this environment is accessible to all children. One such strategy in which environmental change to the classroom can be achieved is through the introduction of sit-stand desks. Unlike traditional classroom desks, sit-stand desks are height adjustable and enable the user to alternate between sitting and standing postures.

Rapid increases in sedentary time have been observed in children aged 11 years and above relative to younger age groups [13, 14]. This trial will target year 5 classrooms, comprising children just below this age (9–10-year-olds), with the goal of reducing the typical rise in sedentary time seen during the transition into adolescence, given sedentary behaviours track from childhood into adolescence and adulthood [9]. Furthermore, evidence suggests that children in year 5 upwards are active participants in their own learning, making them an optimal target for classroom interventions that may facilitate learning [15].

Early-phase studies have demonstrated the feasibility of incorporating sit-stand desks in primary school classrooms over the short term (< 12 weeks) [6, 16, 17]. We have previously observed that sit-stand desks enable pupils (9–10-year-olds) to alternate between sitting and standing, without disruption to teaching, learning or behaviour [6]. International studies have shown sit-stand desks in school classrooms to be effective in increasing energy expenditure [18, 19] and standing and movement [20] during the school day. Other research has shown that sit-stand desks in classrooms lead to improvements in children’s posture and musculoskeletal comfort [21, 22] and levels of academic engagement [23] and achievement [22]. Two studies have assessed the impact on sitting directly, both revealed reductions in children’s daily sitting time of approximately 1 h/day over 4 and 9 weeks follow-up [17, 24]. Recent reviews have concluded that the use of sit-stand desks in school classrooms shows promise; however, the evidence to date is limited to relatively small-scale studies with short intervention periods, and a lack of randomised controlled trials (RCTs) [25, 26]. Furthermore, limited research has examined the impact of this type of intervention on children’s learning and academic achievement [25, 26]. This pilot RCT will build on earlier research by assessing the longer-term acceptability of this low burden environmental intervention in primary schools in the UK, along with the acceptability of a range of health and education-related outcome measures to inform a definitive trial.

### Study aim and objectives

The aim of this study is to undertake a pilot cluster RCT of the introduction of sit-stand desks in primary school classrooms to inform a future fully-powered definitive trial. A definitive trial would examine the impact of the introduction of sit-stand desks over one school year on weekday sitting time (the primary outcome), health and education related outcomes, with sub-group analyses for ethnicity. To inform such a trial, key information needs to be established around school and participant recruitment, acceptability of the intervention and outcome measures, and attrition rates. The objectives of this pilot study are therefore to

1. Establish and refine a recruitment strategy for schools and pupils.

2. Determine attrition in the trial (schools and children).

3. Determine completion rates for outcome measures (and whether these are sufficiently high to provide accurate data in a full trial).

4. Assess whether there are any differences in trial recruitment, retention and acceptability between ethnic groups.

5. Assess the acceptability of randomisation to schools to the intervention or standard practice.

6. Assess the acceptability of measurement instruments to teachers, children and parents, including the activPAL inclinometer as the tool for the measurement of the primary outcome.

7. Assess the acceptability of the intervention to teachers, children and parents.

8. Monitor any adverse effects, such as musculoskeletal discomfort and/or disruption to the classroom environment/teaching and learning to inform the design of a full trial and minimise or eliminate any such effects.

9. Assess intervention fidelity.

10. Derive preliminary estimates of the effect of the intervention on children’s total daily sitting time, physical activity, indicators of health (markers of adiposity and blood pressure), cognitive function, quality of life, and academic performance, engagement and behaviour.

11. Estimate the standard deviation of the primary outcome to inform a sample size calculation for a full RCT.

12. Determine availability and completeness of economic data and conduct a preliminary assessment of potential cost-effectiveness.

## Methods/Design

**Fig. 1:**
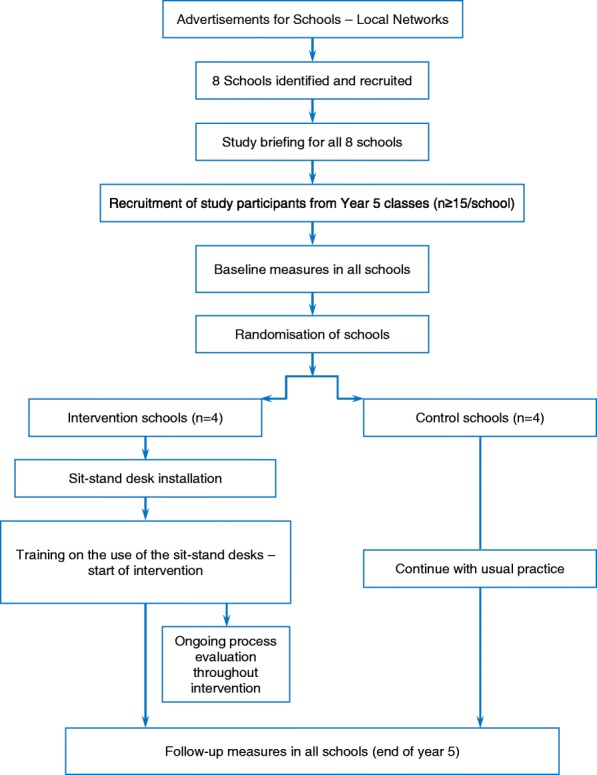
Flow diagram for the Stand Out in Class pilot RCT

**Fig. 2:**
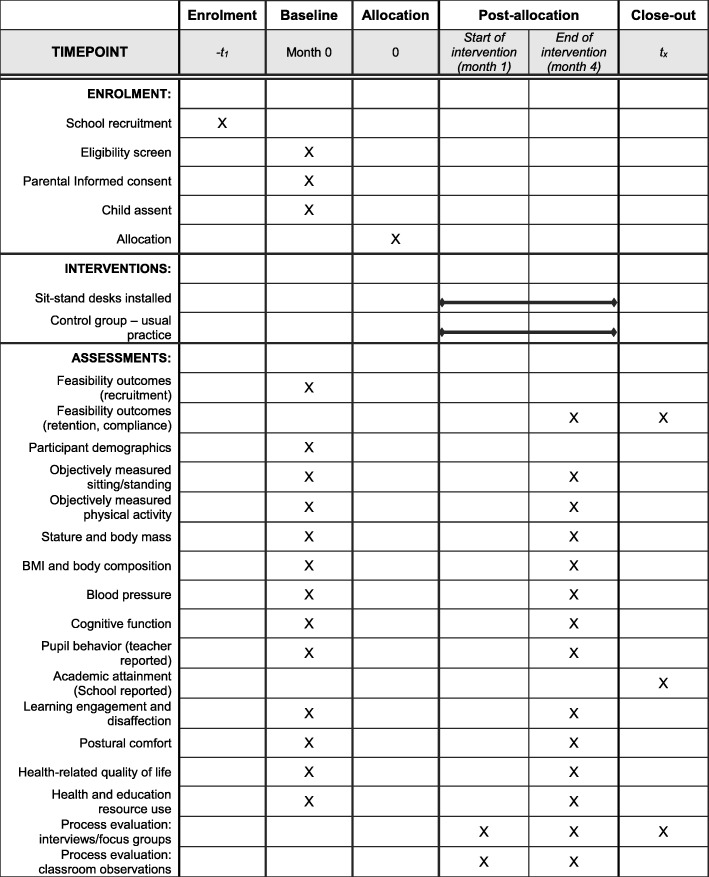
Standard Protocol Items: Recommendations for Interventional Trials (SPIRIT) diagram illustrating the design and timescales of the pilot Stand Out in Class RCT

The design of this study is based on guidance from the UK Medical Research Council for developing and evaluating complex interventions [27]. This is a school-based, pilot two-armed cluster RCT with economic and process evaluations. Individuals (year 5 children, aged 9–10 years) will be the unit of analysis and schools (clusters) randomly assigned to one of two conditions: (1) six manually adjustable sit-stand desks incorporated into the classroom environment (intervention condition), or (2) current practice (control condition). Baseline measurements will precede randomisation. The sit-stand desks will be installed into the intervention classrooms following randomisation. An identical set of outcome measurements will be taken from all participants approximately 6 months after the baseline measures, when participants are at the end of year 5. Observations, interviews and focus groups with teachers, children and parents will be conducted throughout the intervention period as part of a full process evaluation. Figure 1 shows the study flow diagram, and Fig. 2 indicates the schedule of enrolment, intervention and outcome measurements. The project has been granted ethical approval by the Loughborough University Ethical Advisory Committee (reference R16-P027).

### Setting

This study will be conducted in primary schools in Bradford. Bradford was chosen as the study location given its ethnic composition (predominantly South Asian and White British) and high levels of deprivation, health inequalities and childhood morbidity [28]. In 2016–17, 38% of year 6 children in Bradford were overweight or obese according to the National Child Measurement Programme [29]. Half of all babies born in Bradford are of South Asian origin and 60% are born into the poorest 20% of the population [28], with elevated risk factors for chronic diseases seen in South Asian children in comparison to White children [10, 30]. The setting of this study therefore is fundamental in addressing the important issue of health inequalities, in that the intervention will be accessible to all children. In addition, the location enables us to pilot this intervention under challenging circumstances meaning that if it proves acceptable it is likely to be transferable to most schools.

### Sample size

Eight primary schools, each with at least 15 child participants (approximately 50% of a typical class) will be recruited giving a minimum total sample of 120. This sample matches the minimum of four clusters per arm recommended for a cluster RCT [31], and the minimum sample size exceeds the recommendation for pilot trials [32]. Consequently, the sample will be sufficiently large to provide clear estimates of recruitment and follow-up for a definitive trial.

### School recruitment and inclusion criteria

Government funded primary schools located in the City of Bradford will be invited to participate in the study following ethical approval. Private and designated special schools and schools with less than 25 pupils in year 5 will be excluded. Schools will also not be eligible for participation if they are running a sitting time reduction programme, or if they have a unique characteristic that prevents comparison with control schools. The study will be publicised to primary schools through existing local networks (e.g. Bradford Primary Improvement Partnership and Bradford’s Public Forum for Education). We will aim to recruit four schools with predominantly South Asian pupils (> 50%) and four schools with predominantly White British pupils (> 50%). Interested schools will be followed-up via telephone and visits by the research team. Consent will be sought from school management (Head Teacher, Senior Teachers, Governors) and year 5 teachers to participate in the study. During recruitment, schools will be informed they may be randomised to a current practice control condition where they will be asked to maintain their usual classroom practice.

### Participant recruitment and inclusion criteria

All year 5 children (9–10-years-old) within the participating schools will be eligible to participate. Parents will be sent a detailed information sheet about the study so that they can make an informed decision about their child’s participation. They will also be invited to a school meeting outlining the study. Parental/guardian consent (through an opt-in form for their child to participate in the intervention evaluation) will be collected. Children will be asked to provide verbal assent at each data collection time point for the evaluation measures. Children without parental consent for their participation in the evaluation, or those who do not give their assent to participate in the evaluation, will be excluded from the evaluation measures described below. These children will however be able to use the sit-stand desks within their classrooms. Any children in the intervention schools with known contraindications (for example, a musculoskeletal injury, a wheelchair user) that would preclude periods of standing will be invited to participate in the evaluation measures and encouraged to use the sit-stand desk in a seated posture for inclusivity. These individuals will however be excluded from the analyses.

### Allocation to treatment groups

To assess the acceptability of the intervention and proposed outcome measures for use in a definitive trial across an ethnically diverse sample, recruited schools will be stratified based on the ethnic composition of their pupils (identified above). Schools within each stratum will be randomised into the two study arms (intervention and control, using an allocation ratio of 1:1) by an independent statistician at the Leicester Clinical Trials Unit (CTU) following the completion of baseline measurements. Two schools with predominantly South Asian pupils and two schools with predominantly White British pupils will be randomised into the intervention and control arms (four schools in each arm). Stratification of schools will enable us to examine whether there are ethnic differences in terms of participant recruitment and retention, adherence to the outcome measures, and preliminary effects of the intervention on outcome measures. The statistician performing the analyses will be blinded to the schools allocation to the study arms, as will the community researchers undertaking the outcome measurements.

### Experimental intervention

**Fig. 3:**
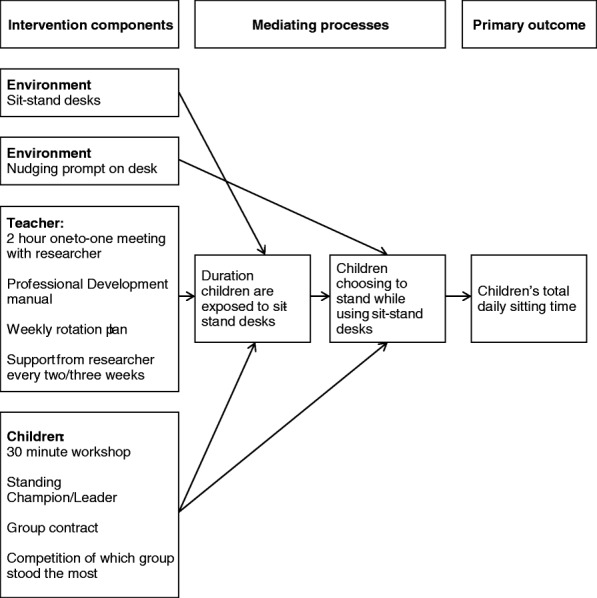
A simplified logic model linking the Stand Out in Class intervention components to hypothesised mediating processes and the primary outcome

Six sit-stand desks will be placed in one year 5 classroom (replacing standard desks) in each intervention school for two school terms. The research team will support teachers in the development of a rotation plan to ensure that all children in their class are exposed to the sit-stand desks for at least 1 h/day on average across the week. Stools or chairs will remain and children will be free to choose whether they sit or stand. Teachers and pupils in the intervention classrooms will receive training on sit-stand desk use by the research team. Teachers will also receive a Professional Development Manual containing information on the health benefits of reducing prolonged sitting and information on correct posture when standing at the desks. The manual and training will focus on encouraging correct adoption of the intervention targeting key barriers and facilitators to sit-stand desk use, identified from our previous work [6, 33] and from the Capability, Opportunity, and Motivation to perform a Behaviour (COM-B) model within the Behaviour Change Wheel [34] and the Theoretical Domains Framework [35] (e.g. self-efficacy, motivation and knowledge). Standardised behaviour change techniques (e.g. goal setting, instruction) [36] will also be used. A summary of the intervention components is shown in Table 1 and Fig. 3 represents a simple logic model for the Stand Out in Class intervention. Additional file 1 details potential intervention barriers, solutions and hypothesised mediating processes informed by the above theoretical frameworks.

**Table 1:** Components of the Stand Out in Class intervention

Intervention component	Target domain	Meditating variable	Description
Adjustable sit-stand desks	Environment	Exposure to desks	Six adjustable sit-stand desks introduced into the classroom
Nudging prompts	Environment	Children choose to stand rather than sit when using desks	Stickers placed upon each of the sit-stand desks • Have you stood up this lesson? • Standing tall and proud this lesson?
2-h one-to-one meeting	Teacher	Exposure to desks	2-h meeting with teacher which will cover: - Why it is important to increase standing/reduce sitting - Importance of exposure to sit-stand desks - Safety—how to use the desks - Rotation plan and example, plus creation of a rotation plan for the following 2 weeks
Professional Development Manual	Teacher	Exposure to desks	Cover topics such as - Why it is important to increase standing/reduce sitting - Importance of exposure to sit-stand desks - Safety—how to use the desks - Rotation plan example
Planned weekly rotation plan	Teacher	Exposure to desks	Teacher creates a predetermined rotation plan and keeps a record of whether this was met or not—simple tick sheet
Fortnightly support with practitioners	Teacher	Exposure to desks	Phone or face-to-face meeting with researchers/practitioners—discuss any issues around implementation of rotation plans.
30-min workshop	Children	Exposure to desks Children choose to stand rather than sit when using desks	Cover topics such as - Why it is important to increase standing/reduce sitting - Importance of exposure to sit-stand desks - Safety—how to use the desks - Rotation plan and how it will work - Discuss other intervention aspects—social contract and competition
Standing champion/leader	Children	Exposure to desks Children choose to stand rather than sit when using desks	One child in a group is chosen as a standing champion with responsibility of reminding the teacher of the rotation plan.
Group contract	Children	Exposure to desks Children choose to stand rather than sit when using desks	Children are all asked to sign a large contract which will state: I will try my best to - Stand up in a good posture when using the sit-stand desk. - Not to disturb the class when I stand up at my sit-stand desk. - Remember when it is my turn to use the sit-stand desk. - Be very careful not to hurt myself or other people when I use the sit-stand desk.

### Control arm

To compare the effects of the intervention against usual practice (i.e. the provision of standard classroom desks), schools assigned to the control arm will be requested to continue with their usual practice and lesson delivery, no environmental changes will be made to their classrooms. The year 5 participants in the control schools will be asked to complete the same study measurements as those in the intervention schools at the same time points. Upon completion of the study, control schools will receive a report summarising their pupils’ sitting and physical activity data. They will also receive adapted materials (i.e. the Professional Development Manual provided to teachers in the intervention schools outlining the benefits of reducing sitting but excluding references to sit-stand desks) upon completion of all follow-up evaluation measures.

### Measurements

#### Trial feasibility-related outcomes

The predominant aim of this pilot study is to establish school and participant recruitment and retention rates, acceptability of the intervention and proposed outcome measures, intervention and measurement fidelity, and the availability and completeness of economic data for an estimation of potential cost-effectiveness in order to inform the development of a fully powered definitive trial. Study uptake will be monitored by recording the number of schools and pupils approached, and the number agreeing to participate (objective 1). Withdrawal rates of schools and children (objective 2) and completion rates for outcome measures will be summarised (objective 3), and these feasibility outcomes will be compared between ethnic groups (objective 4).

#### Process evaluation

A series of interviews and focus groups with teachers, children and parents will be conducted following the completion of baseline measures and randomisation to explore the acceptability of trial procedures, including randomisation (objective 5), and the acceptability of the measurement instruments (objective 6). Eight one-to-one interviews (one per teacher) will be undertaken with the participating year 5 teachers during this phase of the process evaluation, and eight separate focus groups (one per school) will be undertaken with children and parents. We will aim to recruit between four and six participants for each focus group conducted throughout the process evaluation. Acceptability of the intervention (objective 7), and children’s, teacher’s and parent’s perceptions and experiences of the intervention and outcome measures (including any negative effects such as discomfort from the monitoring equipment, reasons for non-compliance to the outcome measures, or class disruption) will be obtained during the intervention through a further set of interviews (*n* = 4, with teachers from the intervention schools) and focus groups (four with children and four with parents from the intervention schools) (objective 8). Differences in trial and intervention acceptability between ethnic groups will be explored as part of the analyses from the focus groups (objective 4). Towards the end of the intervention, four one-to-one interviews will be conducted (one per intervention school) with senior staff (head teachers/deputy head teachers) to further examine the acceptability of the intervention (objective 7). Table 2 provides a detailed plan of the measures and methodologies which will be used in the process evaluation.

**Table 2:** The Stand Out in Class pilot RCT process evaluation plan

Areas to measure	General process questions	Data source and data collection method	Total numbers and sampling strategy
Acceptability of randomisation and measurement tools (objectives 5 and 6)	How did schools feel about being randomised to intervention/control arms? How did schools/children/parents experience recruitment and outcome assessments? Did schools/children/parents find outcome assessments acceptable? What were the reasons for not participating in the trial and/or not complying to the outcome measures?	Interviews with teachers In-class focus groups with children Focus groups with parents Recruitment data (numbers consenting), and missing data from outcome assessments One-to-one interviews with parents and children not participating and/or not complying	Eight teachers from participating schools (end of summer term) Eight focus groups with children (one per school) after baseline assessments. Eight focus groups with parents (one per school) after baseline assessments. Data collected at baseline and follow-up Interviews conducted after baseline assessments
Intervention acceptability and fidelity (objectives 7 and 9)	Was the intervention implemented as planned?	Interviews with intervention teachers Observations of lessons	Four intervention teachers Time-points (T): T1: After desk installation T2: End of year 5 Four intervention schools observed once per term
Intervention acceptability and fidelity (objectives 7 and 9)	What proportion of the target group participated in the intervention?	Teachers logs and telephone interviews with intervention teachers	Four intervention teachers recording use of desks in log book and brief telephone contact every 2 weeks
Intervention acceptability and fidelity—potential moderating factors (objectives 7 and 9)	How were children engaged with sit-stand desks? How satisfied were schools/children/parents with sit-stand desks? How did schools/children/parents perceive the outcomes and usefulness of sit-stand desks	Focus groups with children (in class) Interviews with teachers Focus groups with parents	Four in class focus groups with children from intervention schools: Time points: T1: After desk installation T2: End of year 5 Four intervention teachers T1: After desk installation T2: End of year 5 Four parent focus groups from intervention schools (after desk installation)
Intervention acceptability and fidelity—strategies to facilitate implementation (objective 7 and 9)	What strategies were used to support introduction of standing desks? How were these strategies perceived by staff involved within project?	Interviews with teachers	Four intervention teachers Time-points: T1: After desk installation T2: End of year 5
Intervention acceptability and fidelity—quality of delivery (objectives 7 and 9)	How well were sit-stand desks introduced? What is the quality of the sit-stand desks and professional manual?	Interviews with teachers Focus groups with children (in class)	Four intervention teachers Time-points: T1: After desk installation T2: End of year 5 4 in class focus groups with children from intervention schools: T1: After desk installation T2: End of year 5
Intervention fidelity—context (objective 9)	What factors at political, economical, organisational and work group levels affected the implementation?	Interviews with teachers and head teachers	Four intervention teachers (end of year 5) Four interviews with head teachers from intervention schools (end of year 5)

To assess intervention, fidelity intervention classrooms will be observed by a member of the research team for a duration of at least half a school day during the Spring and Summer terms. These classroom observations will take place once per school term (Spring and Summer) for each intervention school (eight observations in total, two per intervention school). During these observations, every 10 min the researcher will record the number of boys and girls who are sitting or standing when using the sit-stand desks. Field notes will also be taken to document the occurrence of any intervention components (i.e. use of prompt cards, engagement with a standing champion, see Table 1) during the observation period. Children’s posture during sit-stand desk use will also be recorded using a postural analysis recording system based on the Portable Ergonomic Observation (PEO) [21] to assess any future risk of musculoskeletal injury (objectives 8 and 9).

#### Health- and education-related outcome measures

All health-related outcome measurements will be taken twice, at baseline prior to randomisation, and approximately 6 months after baseline when pupils are at the end of year 5. The likely primary outcome in a definitive trial would be change in average daily school-day sitting time. Sitting will be measured objectively for seven consecutive days during each measurement period using the activPAL3 micro accelerometer. The activPAL3 will be waterproofed (using a nitrile sleeve and hypoallergenic Hypafix [BSN Medical] dressing) and participants will be requested to wear the device continuously (24 h/day) on the anterior aspect of their right thigh during each measurement period. Participants will be provided with a brief diary during each monitoring period where they will be requested to document time in bed and any periods of non-wear.

The use of the activPAL to objectively measure sitting has increased in recent years [37], and the device has been successfully used as a primary outcome measure in previous school-based sedentary behaviour interventions [6, 17, 24] and is recommended for use in interventions when the primary outcome measure is sitting [38]. It is regarded as the most accurate method of assessing sitting behaviour in free-living settings [39] and has been shown to be almost 100% accurate in measuring sitting, standing, walking and postural transitions in children [40, 41]. Whilst total daily school-day sitting time is likely to be the primary outcome for a future trial, we will also extract classroom and leisure-time sitting, standing and stepping time, along with the number of transitions between sitting and standing from the activPAL data. Periods of non-wear and sleep time will be excluded from the analyses using an automated algorithm [42], supplemented with cross-checking against participant’s diary entries. We will examine any positive (i.e. reductions in sitting) or compensatory effects (increases in sitting) of the intervention on children’s behaviour out of school hours. The variability (standard deviation) of the data from the proposed primary outcome will be used to inform a sample size calculation for a definitive trial (objective 11).

Proposed secondary outcomes for a definitive trial include objectively measured physical activity. While the activPAL provides a valid measure of posture, it has not been well validated for assessment of various physical activity intensities among children. Therefore, children will wear the ActiGraph GT3X+ accelerometer on the waist continuously (24 h/day) for seven consecutive days, concurrently with the activPAL, during each measurement period. Periods of non-wear will be documented in a brief diary provided with the devices. Waist worn accelerometers have traditionally been considered the criterion measure of children’s physical activity [43]. The ActiGraph is the most commonly used accelerometer in field-based research and has been shown to have acceptable reliability and validity in paediatric populations [44]. Times spent in light (26–573 counts per 15 s epoch) and moderate-to-vigorous intensity (≥ 574 counts per 15 s epoch) activity throughout the day, and during and out of school hours will be extracted from the ActiGraph data using the Evenson cut-points [45]. In a comparative study examining the classification accuracy of five different ActiGraph cut-points for determining children’s and adolescent’s physical activity intensity, against indirect calorimetry, the Evenson cut-points were highlighted as the most accurate across all intensity levels and are therefore recommended for use in this age group [46]. Periods of non-wear and sleep time will be excluded from the analyses during the processing of the ActiGraph data, supplemented with cross-checking against participant’s diary entries.

At each measurement point, children’s stature and body mass (both assessed without shoes) will be measured directly using standard procedures by trained research staff and BMI will be calculated and converted to a BMI percentile based on UK reference data [47]. Body composition (percentage body fat and fat mass) will be assessed via bio-impedance analysis, using Tanita DC-360S body composition scales which contain specific algorithms for children. Blood pressure will be measured from the left arm after at least a 5-min period of quiet sitting using a semi-automated recorder (Omron HEM-907) with a paediatric cuff, in accordance with current recommendations [48]. Three measurements of blood pressure will be taken; each measurement will be separated by a 2-min rest period. The mean systolic and diastolic blood pressures recorded from the second and third assessments will be calculated and used in the analyses.

A set of objective cognitive function tests will also be administered via a validated software package. The software will be installed on school computers enabling a group of students (those with parental consent to participate in the intervention evaluation) to undertake these assessments at the same time in the classroom under the supervision of two researchers. Participants will undertake a practise run through of the cognitive function test battery a day before the test day. The cognitive function test battery will take children approximately 15 min to complete and will include the Corsi block tapping test [49], the Stroop test [50] and the rapid visual information processing (RVIP) task [51]. The Corsi block tapping test is a measure of visuo-spatial working memory capacity [49]. Performance on the Corsi block tapping test is linearly associated with age in typically developing children [52], and the original version has exhibited a test-retest reliability coefficient of 0.7 (Pearson’s correlation coefficient) in 11-year-olds [53]. The Stroop test measures sensitivity to interference and the ability to suppress an automated response (reading colour names in favour of naming the font colour); it is a commonly used measure of selective attention and executive function [54]. The complex reaction time measure within the computerised Stroop test has been shown to exhibit a test-retest reliability of 0.55 (Pearson’s correlation coefficient) in a combined sample of children and adults [55]. The RVIP is a measure of sustained attention and has been shown to exhibit an internal reliability coefficient (assessed using Cronbach’s alpha) of 0.49 in school-age children [56].

Measures of pupils’ academic progress and attainment will be collected using routine assessment data collected by the schools at half-termly intervals. The impact of the intervention on pupil’s behaviour will be assessed using the Strengths and Difficulties questionnaire completed by teachers at baseline and follow-up. This questionnaire, when completed by teachers, has been shown to provide a valid indicator of children’s behaviour (convergent validity: Pearson’s correlation coefficient with the Rutter questionnaire = 0.92 [57]). Children will also report their engagement and disaffection with their own learning [58] (correlation coefficients between pupil and teacher reports of the components of engagement using this measure range from 0.26 to 0.44 [59]) and their postural comfort [21]. In addition, the Paediatric Quality of Life Inventory (PEDS-QL) [60] and EuroQol 5-dimension Youth (EQ-5D-Y) [61] will be completed by children to provide a measure of self-reported quality of life at each measurement point. The construct validity of the PEDS-QL has been previously demonstrated with healthy children displaying significantly higher scores on this measure in comparison to acutely or chronically ill children [60]. Responses on the EQ-5D-Y have been shown to correlate with other measures of children’s health-related quality of life (convergent validity correlation coefficients up to 0.56 [62]). To further inform the economic analysis (objective 12), teachers and parents will also complete a questionnaire (created for the purpose of this study) assessing participants’ health- and education-related resource use at baseline and follow-up. Basic demographic information (sex, age, ethnicity and postcode to determine Index of Multiple Deprivation (IMD) as an indicator of socio-economic status) will be collected at baseline.

### School and participant appreciation

As a thank you for participating in the pilot trial, all schools (intervention and control) will receive a donation of £200 at the end of the trial. A £5 gift voucher will be given to children following the completion of both baseline and follow-up measures to encourage a timely return of the accelerometers.

### Economic analysis

The availability and completeness of economic data will be established as part of this pilot study (objective 12). Resource-use information will be collected, which will include the cost of the sit-stand desks, along with participants’ health- (e.g. General Practitioner visits) and education-related resource use (e.g. requirements for additional tutoring). Proposed outcomes within a definitive trial will be based on two sectors, health and education. For the former we will use the PEDS-QL [60] and EQ-5D-Y [61] delivered at baseline and follow-up to assess children’s health-related quality of life; for the latter, we will separately present measures of academic performance obtained through the schools’ routine assessment data. A preliminary cost-effectiveness analysis will be conducted to inform the value of a full trial and to make recommendations for the design of the full trial. We will present cost and PEDS-QL and EQ-5D-Y data as an incremental cost-effectiveness ratio where the additional cost associated with the provision of sit-stand desks is formally compared with the additional benefit of providing sit-stand desks. We will also present a comparison of pupils’ academic achievement in each group. A brief scoping review will be carried out to identify existing model(s) that link the shorter-term outcomes to longer-term effects (on both health and education outcomes [63]) and where feasible, we will use these model(s) to examine the likely additional costs and benefits of the intervention over the longer term.

### Data analysis

#### Statistical analysis

This study will be analysed and reported according to the Consolidation Standards of Reporting Trials (CONSORT) statement for cluster RCTs [64]. Data will be analysed on a complete-case basis. The purpose of the primary analysis is to assess the feasibility of recruitment and adherence/retention of primary schools and pupils to a sit-stand desk intervention. As this is a pilot trial, the primary analyses will mainly utilise descriptive statistics. We will summarise the number of schools approached, the number agreeing to participate, the proportion of children within each school with parental consent to participate in the study evaluation, the number of children completing the study protocol, retention rates, and the number providing valid outcome measurement data at baseline and follow-up. The study acceptability data will be presented for the sample as a whole and stratified according to study arm (intervention and control) and ethnicity (South Asian and White British).

While the main aim of this study is to establish acceptability, feasibility, recruitment rates and sample size to inform a definitive trial, and although effectiveness will unlikely be established with the small sample size, we will examine the primary and secondary outcomes to mimic practice for a full trial. Results from this analysis will be treated as preliminary and interpreted with caution [65, 66]. As the number of clusters is low, cluster summary statistics will be used rather than multi-level modelling [67, 68]. The analysis will be carried out using children as the unit of analysis with change in average total school-day sitting time as the primary outcome. A weighted linear regression model will be used to compare the intervention arms weighted by the number of participants followed up in each cluster and adjusted for baseline total daily sitting time on school days for each cluster. To examine preliminary effects of the intervention on the secondary outcomes, the same analytical approach will be adopted as for the primary outcome (objective 10). These analyses will provide preliminary evidence of the effectiveness of the intervention and will in-part inform the decision of whether a definitive trial should be undertaken.

Sensitivity analyses will be performed on the proposed primary outcome for a full trial of average weekday sitting time, and on the secondary activPAL (standing and stepping time) and ActiGraph (time in light physical activity and moderate-to-vigorous physical activity) variables. This will be achieved by including pupils who have worn the activPAL and ActiGraph (with a minimum valid wear time of 8 h each day) for at least 1, 2, 3 and 4 weekdays, and on at least 2 weekdays and 1 weekend day at both baseline and follow-up.

The objective of this pilot study is to estimate the standard deviation of the primary outcome to inform a full trial. The intraclass correlation coefficient (ICC) to inform the sample size calculation of the definitive trial will be estimated from published literature. The ICC will not be estimated from this pilot study, because multilevel modelling is considered to be an unsuitable analysis method in the present study due to low number of clusters and an ICC estimate from such a model would not be sufficiently robust to inform a sample size calculation for a definitive trial.

#### Qualitative analyses

Audio-recordings of interviews and focus groups with teachers, parents and children collected as part of the process evaluation will be transcribed verbatim and analysed using framework analysis [69, 70], using the Normalisation Process Theory [71] and the Theoretical Domains Framework [35] as the underpinning theoretical framework. These will be supplemented with field notes from observation sessions. Collectively, these will provide information on the acceptability of the trial procedures including randomisation, the measurement instruments and the overall acceptability of the intervention.

### Data management and research governance

Anonymised data will be entered into a secure and validated clinical data management system provided by the Leicester CTU; this database (InferMed Macro v4) includes a series of quality control mechanisms to ensure that the data collected are complete and accurate. The study will be sponsored by Loughborough University. Two groups will be created to oversee the study; an independent Trial Steering Committee (TSC) and a Project Committee. As the study is regarded as low risk, the TSC will take on the role of a Data Monitoring Committee and review any serious adverse events which are thought to be intervention related and monitor progress with data collection. The TSC will meet every 6 months and include the principle investigators (Clemes and Barber), an independent chair, two independent external members (including a statistician) and two school representatives. The Project Committee will comprise the principle investigators, all co-investigators and those concerned with the day to day running of the study. The Project Committee will meet monthly and provide an update report for the TSC. Additional file 2 details the Standard Protocol Items: Recommendations for Interventional Trials (SPIRIT) checklist for this protocol paper.

## Discussion

The pathogenesis of chronic diseases such as type 2 diabetes and cardiovascular disease have origins in childhood [72]. The precursors of chronic disease risk in young people are a major public health issue, given links between lifestyle behaviours and disease risk. Sedentary behaviour is highly prevalent in children and adversely associated with adiposity/weight gain and clustered metabolic risk [7, 73]. As sedentary behaviours track from childhood into adulthood [9], reducing children’s sitting time could reduce the risk of numerous chronic conditions linked to prolonged sitting in adulthood, including obesity, some cancers, type 2 diabetes and cardiovascular disease mortality [3, 4, 74].

The need for more, and more effective, interventions targeting the primary prevention of chronic diseases by encouraging healthy lifestyles in children has been explicitly acknowledged. The Chief Medical Officer for England’s 2012 Annual Report, ‘Our Children Deserve Better: Prevention Pays’ and the UK’s ‘Childhood Obesity Plan for Action’ highlight the importance of the school environment in promoting healthy behaviours [75, 76]. While there are examples of school-based sedentary behaviour interventions from Australia [77], New Zealand [17, 24] and the USA [19, 23], differences in school systems, curricula and cultures preclude direct translation of these findings to the UK. Schools are a crucial setting for addressing health inequalities, with classroom-based interventions being accessible to all children. The ethnically diverse and socio-economically deprived geographical location of this study allows us to test this type of intervention under challenging circumstances. If acceptable, it is likely to be transferable to most UK schools.

This pilot trial will provide the detailed information and insight needed to design a definitive cluster RCT (if preliminary estimates of effectiveness are observed), which will examine the impact of sit-stand desks in the classroom environment on health- and education-related outcomes in UK primary school children.

## Additional files

Additional file 1: Potential intervention domain barriers and solutions to barriers using the Capability, Opportunity, Motivation to perform a Behaviour model (COM-B), Theoretical Domains Framework (TDF) and behaviour change techniques (BCT). (DOCX 45 kb)

Additional file 2: SPIRIT checklist. (DOCX 51 kb)

## Abbreviations

BMI: Body mass index
COM-B model: Capability, Opportunity, and Motivation to perform a Behaviour model
CONSORT: Consolidation Standards of Reporting Trials
CTU: Clinical trials unit
EQ-5D-Y: EuroQol 5-dimension Youth
ICC: Intraclass correlation coefficient
IMD: Index of Multiple Deprivation
METs: Metabolic equivalents
PEDS-QL: Paediatric Quality of Life Inventory
PEO: Portable Ergonomic Observation
RCT: Randomised controlled trial
RVIP: Rapid visual information processing task
SPIRIT: Standard Protocol Items: Recommendations for Interventional Trials
TSC: Trial Steering Committee
UK: United Kingdom
