# Supplementary material for: Stand Out in Class: restructuring the classroom environment to reduce sedentary behaviour in 9–10-year-olds — study protocol for a pilot cluster randomised controlled trial
Source: Pilot Feasibility Stud. 2018 May 24;4:103. doi: 10.1186/s40814-018-0295-3 (PMC5966899; doi:10.1186/s40814-018-0295-3)
Supplement: Supplementary file 1 — Potential intervention domain barriers and solutions to barriers using the Capability, Opportunity, Motivation to perform a Behaviour model (COM-B), Theoretical Domains Framework (TDF) and behaviour change techniques (BCT). (DOCX 45 kb) [file 40814_2018_295_MOESM1_ESM.docx]

| **Additional file 1.** Potential intervention domain barriers and solutions to barriers using the Capability, Opportunity, Motivation to perform a Behaviour model (COM-B), Theoretical Domains Framework (TDF) and Behaviour Change techniques (BCT). | | | | | | |
| --- | --- | --- | --- | --- | --- | --- |
| **Barriers** | **Affected meditating (M) variable** | **COM-B** | **TDF** | **Solution**  **(What, Who, How, Where)** | | **BCT** |
| **Environmental barriers** | | | | | | |
| Lesson activities may require a large amount of space (e.g. A3 poster) and for children to work in groups (cluster of tables, e.g. 6 children) which may be difficult using the sit-stand desks which seat individual children, not the standard pair of children, some children may stand, some may sit; therefore sit-stand desks maybe seen as impractical for the teacher, and the teacher could tell the children using the six sit-stand desks to sit, not stand. | M2) Children choosing to stand when using sit-stand desks. | Opportunity –physical | Environmental contexts and resources | **What:** 1) Training manual; 2) one-to-one training; 3) Frequent support conversations.  **Who:** Researchers/practitioners for all three components  **Where/how:** 1 & 2 – school; 3 – school or via telephone  **When:** 1 & 2 – before the desks are introduced into the classroom. 3 every two weeks throughout the intervention. | | 4.1. Instruction on how to perform the behaviour.  5.1. Provide information about health consequences  12.1. Restructuring the physical environment  3.1. Social support (unspecified) |
| Limited classroom size with six sit-stand desks in place. | M1) Time children are exposed to sit-stand desks. | Opportunity – physical | Environmental contexts and resources | **What:** 1) Training manual; 2) one-to-one training; 3) Frequent support conversations.  **Who:** Researchers/practitioners for all three components  **Where/how:** 1 & 2 – school; 3 – school or via telephone  **When:** 1 & 2 – before the desks are introduced into the classroom. 3 every two weeks throughout the intervention. | | 4.1. Instruction on how to perform the behaviour.  5.1. Provide information about health consequences  12.1. Restructuring the physical environment  3.1. Social support (unspecified) |
| Limited flexibility of the sit-stand desks for the teacher to ask the children to work in groups. (traditionally would be around their cluster of tables.) | M1) Time children are exposed to sit-stand desks. | Opportunity – physical | Environmental contexts and resources | **What:** 1) Training manual; 2) one-to-one training; 3) Frequent support conversations/ consultation.  **Who:** Researchers/practitioners for all three components  **Where/how:** 1 & 2 – school; 3 – school or via telephone  **When:** 1 & 2 – before the desks are introduced into the classroom. 3 every two weeks throughout the intervention. | | 3.1. Social support (unspecified) |
| Teacher might want to place the children with special needs (e.g. eye sight problem or misbehaviour) closer to the front or a specific place in the class room. | M1) Time children are exposed to sit-stand desks. | Opportunity – physical | Environmental contexts and resources | **What:** 1) Training manual; 2) one-to-one training; 3) Frequent support conversations/ consultation.  **Who:** Researchers/practitioners for all three components  **Where/how:** 1 & 2 – school; 3 – school or via telephone  **When:** 1 & 2 – before the desks are introduced into the classroom. 3 every two weeks throughout the intervention. | | 3.1. Social support (unspecified) |
| **Teacher’s barriers** | | | | | | |
| Not understanding the importance of reducing children’s sitting/sedentary time, therefore not rotating children and discouraging standing during lessons. | M1) Time children are exposed to sit-stand desks. | Capability – psychological  Motivation-reflective | Knowledge | **What:** 1)Training manual; 2) one-to-one training  **Who:** Researchers/practitioners for all three components  **Where/how:** School  **When:** Before the desks are introduced into the classroom throughout the intervention. | 5.1. Information about health consequences | |
| Find it difficult to rotate the children in groups because of some of the needs of the children or because of challenging behaviour of children | M1) Time children are exposed to sit-stand desks. | Capability – psychological | Cognitive skills | **What:** 1)Training manual; 2) one-to-one training  **Who:** Researchers/practitioners for all three components  **Where/how:** School - face-to-face  **When:** Before the desks are introduced into the classroom throughout the intervention.  **Notes**: during training researchers will aid the teachers in identifying possible challenging children, and then one of the activities will be a practice and rehearsal in how to deal with challenging behaviour. | 8.1. Behavioural practice/rehearsal | |
| After a period of time the teacher does not have belief in the sit-stand desks and ceases to rotate the groups. | M1) Time children are exposed to sit-stand desks. | Motivation – reflective | Belief about capabilities | **What:** Frequent support conversations.  **Who:** Researchers/practitioners  **Where/how:** School or via telephone  **When:** Every two weeks throughout the intervention.  **Notes**: Researcher will reassure the teacher they can successfully do this, and describe the occasions when they did rotate the children successfully.. | 15.1. Verbal persuasion about capability.  15.3. Focus on past success | |
| Rotating the children is difficult and teacher stops rotating because of the added stress and consideration of how to rotate children. | M1) Time children are exposed to sit-stand desks. | Motivation – automatic  Capability- psychological | Emotion | **What:** Frequent support conversations.  **Who:** Researchers/practitioners  **Where/how:** School or via telephone  **When:** Every two weeks throughout the intervention.  **Notes**: Researcher will advise on ways to reduce negative emotions to facilitate the rotating of children to the desks; and also advise on/arrange/provide emotional support – e.g. ask for class-room assistant to attend support conversation – this will be a face-to-face meeting. | 11.2. Reduce negative emotions.  3.2. Social support (practical) | |
| The extra time burden during times of stress and time pressure. | M1) Time children are exposed to sit-stand desks. | Motivation – automatic  Capability- psychological | Emotion | **What:** Frequent support conversations.  **Who:** Researchers/practitioners  **Where/how:** School or via telephone  **When:** Every two weeks throughout the intervention.  **Notes**: Researcher will advise on ways to reduce negative emotions to facilitate the rotating of children to the desks; and also advise on/arrange/provide emotional support – e.g. ask for class-room assistant to attend support conversation – this will be a face-to-face meeting. | 11.2. Reduce negative emotions.  3.2. Social support (practical) | |
| If the rotation system goes against other grouping systems used in class e.g. ability or split class system | M1) Time children are exposed to sit-stand desks. | Capacity – psychological/  physical | Memory, attention and decision processes | **What:** 1) Training manual; 2) one-to-one training; 3) Frequent support conversations.  **Who:** Researchers/practitioners for all three components  **Where/how:** 1 & 2 – school; 3 – school or via telephone  **When:** 1 & 2 – before the desks are introduced into the classroom. 3 every two weeks throughout the intervention. | 4.1. Instruction on how to perform the behaviour.  12.1. Restructuring the physical environment  3.1. Social support (unspecified) | |
| Teacher forgets to rotate children | M1) Time children are exposed to sit-stand desks. | Capacity – psychological | Memory, attention and decision processes | **What:** Children’s workshop. In the children’s workshop, children will be informed of the rotation plan which will be printed and placed on display in the classroom. ‘Remind teacher’ or something similar will be written in large letters next to the rotation-plan display.  **Who:** Researchers/practitioners ensure the time table has been printed off, and ensure children and staff (teacher, assistant) know where the plan is kept and why.  **Where/how:** School  **When:** During the children’s workshop. | 7.1. Prompts/cues | |
| Find it difficult to change the standard sitting dynamics during lessons | M1) Time children are exposed to sit-stand desks. | Motivation – reflective | Belief about capabilities | **What:** Frequent support conversations.  **Who:** Researchers/practitioners  **Where/how:** School or via telephone  **When:** Every two weeks throughout the intervention.  **Notes**: Researcher will reassure the teacher they can successfully do this, change the groupings and rotation plan, or position of desks if required. Also describe to the teacher the occasions when they did rotate the children successfully. | 15.1. Verbal persuasion about capability.  15.3. Focus on past success  4.1. Instruction on how to perform the behaviour. | |
| Teachers will often have children in ability tables and this may disrupt the workings of that, therefore will not rotate children, or rotate children equally. | M1) Time children are exposed to sit-stand desks. | Motivation – reflective | Belief about capabilities | **What:** Frequent support conversations.  **Who:** Researchers/practitioners  **Where/how:** School or via telephone  **When:** Every two weeks throughout the intervention.  **Notes**: Researcher will reassure the teacher they can successfully do this, change the groupings and rotation plan, or position of desks if required. Also describe to the teacher the occasions when they did rotate the children successfully. | 15.1. Verbal persuasion about capability.  15.3. Focus on past success  4.1. Instruction on how to perform the behaviour. | |
| Teacher is absent, so rotation plan is not followed. Or if there is a change in personnel (e.g. part-time teacher, trainee teacher, supply teacher or different teacher for certain classes), there may be inconsistent delivery of the intervention and rotation policy | M1) Time children are exposed to sit-stand desks. | Capacity – physical | Social support | **What:** Children’s workshop. Children will be informed of the rotation plan which will be printed and placed on display in the classroom. ‘Remind teacher’ or something similar will be written in large letters next to the rotation-plan display.  **Who:** Researchers/practitioners ensure the timetable has been printed off, and ensure children and staff (teacher, assistant) know where the plan is kept and why.  **Where/how:** School  **When:** During the children’s workshop. | 7.1. Prompts/cues | |
| Teacher does not have the confidence to manage the group with a proportion of the students standing. | M1) Time children are exposed to sit-stand desks. | Capacity-physical/ psychological | Skills; beliefs about capabilities | **What:** 1) teacher would be supported by the researchers/practitioners planning the rotation; 2) Children will be asked to agree on the rules of using sit-stand desks  **Who:**  Researchers/practitioners  **Where/how:** School  **When:** 1 Teacher’s training; 2 Before the desks are introduced into the classroom (Children’s workshop) | 4.1. Instruction on how to perform the behaviour. | |
| After a period of time the novelty of the intervention has worn off, leading to reduced compliance or reduced enthusiasm by the teacher to encourage standing. | M1) Time children are exposed to sit-stand desks. | Motivation-reflective | Optimism; Belief about capabilities | **What:** Consulting  **Who:** Researchers/practitioner  **Where/how:** Frequently (fortnightly) contact with the teacher via telephone/email and visit in person (at school)  **When:** During the period of intervention | 15.1. Verbal persuasion about capability.  15.3. Focus on past success  4.1. Instruction on how to perform the behaviour. | |
| Teacher does not have a clear goal for the time each of the children’s groups should be exposed to the desks | M1) Time children are exposed to sit-stand desks. | Capability | Skills | **What:** 1) teacher’s manual; 2) one-to-one teacher's training; 3) consulting  **Who:** Researchers/practitioners and teachers  **Where/how:** 1 & 2 school; 3 telephone/email/in person meeting  **When:** 1 & 3 during the period of intervention; 2 at teacher’s training | 8.1. Behavioural practice/rehearsal | |
| Resistant to change - may require a change in teaching practice | M1) Time children are exposed to sit-stand desks. | Motivation | Skills | **What:** 1) one-to-one teacher's training; 2) consulting  **Who:** Researchers/practitioners and teachers  **Where/how:** 1 school; 2 telephone/email and visit in person (at school)  **When:** 1 at teacher’s training; 2 during the period of intervention | 8.1. Behavioural practice/rehearsal  8.7. Graded tasks | |
| **Children barriers** | | | | | | |
| Children do not understand the importance of standing while working; therefore will conform to the usual behaviour of sitting to work when using the sit-stand desks. | M2) Children choosing to stand when using sit-stand desks | Capability – psychological | Knowledge | **What:** Work shop for children  **Who:** Researchers/practitioners  **Where/how:** School for 30-40 minutes  **When:** Before the desks are introduced into the classroom (Children’s workshop) | 4.1. Instruction on how to perform the behaviour.  5.1. Provide information about health consequences | |
| As standing is optional, some children might just choose to sit down from the start. This may affect the attitude of other children in the classroom, and cause less children to opt to stand. | M2) Children choosing to stand when using sit-stand desks | Motivation – reflective | Beliefs about consequences | **What:** Work shop for children  **Who:** Researchers/practitioners  **Where/how:** School for 30-40 minutes  **When:**  Before the desks are introduced into the classroom (Children’s workshop)  **Notes:** Ask children to list pros and cons of standing up more during workshop. Put the list up in the classroom. | 9.2. Pros and cons  7.1. Prompts/cues | |
| Children may not relate the health benefits of standing to their own health. | M2) Children choosing to stand when using sit-stand desks | Motivation – reflective | Beliefs about consequences | **What:** Work shop for children  **Who:** Researchers/practitioners  **Where/how:** School for 30-40 minutes  **When:** Before the desks are introduced into the classroom (Children’s workshop)  **Notes:** Make it clear that sitting down is fine, but try to stand if possible as this is great for your health. | 5.1. Provide information about health consequences  9.2. Pros and cons  7.1. Prompts/cues | |
| Children may not ‘take to it’ first time. This may take children out of their comfort zone and could make some children physically uncomfortable. Some children may require time to get used to standing in class. | M2) Children choosing to stand when using sit-stand desks | Capability – physical | Physical skills | **What:** Work shop for children  **Who:** Researchers/practitioners  **Where/how:** School for 30-40 minutes  **When:** Before the desks are introduced into the classroom (Children’s workshop)  **Notes:** Make it clear that sitting down is fine, but try to stand if possible as this is great for your health. Set the goal of standing for the first 10 minutes each time they work at the table. Include a prompt sticker for children to stand at least for the first 10 minutes. | 5.1. Provide information about health consequences  8.3. Habit formation  7.1. Prompts/cues | |
| Some children may feel self-conscious if they are the only ones who choose to stand. Once other children sit down, they may feel obliged to do the same due to social constraints. | M2) Children choosing to stand when using sit-stand desks | Opportunity – social | Social influences | **What:** 1) Work shop for children; 2) teacher awards a standing champion weekly; 3)Update of Teacher capacity to do this via frequent support conversations  **Who:** 1 & 3 Researchers/practitioners; 2 teachers  **Where/how:** 1 School for 30-40 minutes; 2 School; 3 School or phone call.  **When: 1)** Before the desks are introduced into the classroom; 2 Weekly, 3 every two weeks.  **Note:** Make it clear that even if the child is the only one standing, they are a role model of someone who is working but also being healthy by reducing the time they are sitting. The teacher rewards a standing champion for each week. The child is given a badge and can wear for a week. Clear criteria for standing champion is 1) using the desks to stand up safely and sensibly 2) Length of time is not the most important thing, it’s having an attempt. | 13.1. identification of self as role model  10.3. Non-specific reward | |
| After a period of time the novelty of standing while working has worn off and children go back to sitting when using the desks. | M2) Children choosing to stand when using sit-stand desks | Motivation – automatic | Reinforcement | **What:** Teacher informs researcher (researcher asks)  **Who:** Researchers/practitioners and teachers  **Where/how:** School face-to-face meeting or phone call.  **When:** During the intervention  **Note:** During the teacher fortnightly meetings researchers asks about novelty wearing off. If this is the case ask teachers to either 1) change stickers on desks 2) move the desks to a different physical position; 3) change the groupings of children; 4) change the rotation plan – this all done with the support of the researcher. | 7.1. Prompts/cues  12.1. Restructuring the physical environment.  12.2. Restructuring the social environment. | |
| Children might have very short period of time or no time to move their belongs (stationary/workbooks/etc.) for the rotaion. | M1) Time children are exposed to sit-stand desks. | Capability-physical | Environmental Context and resources | **What:** 1) Children will be asked to rotate during the lunch time, which will give them more time to move their belongings. 2) Storage basket of personal belongingss can be provided if required.  **Who:** Researchers/Practitioners  **Where/how:** School  **When:**  Before the desks are introduced into the classroom (Children’s workshop) | 12.2. Restructuring the social environment  12.1. Restructuring the physical environment | |
| Children find standing is tiring | M2) Children choosing to stand when using sit-stand desks | Capability- physical | Knowledge | **What:** Children will be taught how to stand in a good posture. They will also be told they could “break up their standing time by sitting down”  **Who:** Researchers/Practitioners with children  **Where/how:** School  **When:**  Before the desks are introduced into the classroom (Children’s workshop) | 4.1. Instruction on how to perform the behaviour.  5.1. Provide information about health consequences | |
